# Development and validation of a social cognitive theory-based survey for elementary nutrition education program

**DOI:** 10.1186/s12966-015-0206-4

**Published:** 2015-04-09

**Authors:** Elisha Hall, Weiwen Chai, Wanda Koszewski, Julie Albrecht

**Affiliations:** Department of Nutrition and Health Sciences, University of Nebraska-Lincoln, 110 Leverton Hall, Lincoln, NE 68583 USA; Nutrition and Dietetics, University of North Dakota, Grand Forks, USA

**Keywords:** Validation, Social cognitive theory, Behavior, Self-efficacy, Knowledge, Healthy eating, Elementary nutrition education

## Abstract

**Background:**

The Social Cognitive Theory (SCT) is a widely used model for developing elementary nutrition education programs; however, few instruments are available to assess the impact of such programs on the main constructs of the SCT. The purposes of this study were: 1) to develop and validate a SCT-based survey instrument that focuses on knowledge, behavior, and self-efficacy for fifth grade students; 2) to assess the relationships between knowledge, behavior, and self-efficacy; and 3) to assess knowledge, behavior, and self-efficacy for healthy eating among the fifth grade students.

**Methods:**

A 40-item instrument was developed and validated using content validity and tested among 98 fifth grade students for internal consistency reliability. Relationships between knowledge, behavior, and self-efficacy were assessed using Pearson Correlation Coefficients. Differences in behavior and knowledge scores between children with high and low self-efficacy were examined using t-test.

**Results:**

Cronbach’s alphas for self-efficacy (0.70) and behavior (0.71) subscales of the survey were acceptable, although lower for knowledge (0.56). Summary scores for self-efficacy and behaviors were positively correlated (r = 0.40, P = 0.0001); however, summary knowledge scores were not associated with self-efficacy (r = 0.02, P = 0.88) or behavior scores (r = 0.14, P = 0.23). Participants with high self-efficacy also had significantly higher scores on consuming fruits (P = 0.0009) and dairy products (P = 0.009), eating breakfast (P = 0.008), helping plan family meals (P = 0.0006) and total behaviors for healthy-eating (P = 0.001) compared to those with low self-efficacy. In addition, approximately two thirds of the fifth grade students reported that they did not eat any fruits or vegetables or ate them only once on a typical day.

**Conclusions:**

The developed instrument is a reliable and useful tool to assess SCT-based elementary nutrition education programs, particularly for self-efficacy and behavior. Our results also indicated that strategic interventions are necessary to improve dietary behaviors regarding fruit and vegetable consumptions among elementary school students.

## Background

Childhood obesity is a serious issue in the United States that affects approximately 17% of youth ages 2–19 years old [1]. These rates are concerning for the current generation due to the many health complications that can affect these children, and have the potential to lead into adulthood [2,3]. The school environment is one of the main target areas for children to develop healthy behaviors. Various interventions have been conducted within schools, focusing on nutrition and/or physical activity.

The Social Cognitive Theory (SCT) is one of the widely used models for developing such programs. This theory emphasizes that human behavior depends on the reciprocal interaction of personal, behavioral, and environmental factors [4]. Several interventions grounded in the SCT among 4–6 year old children have demonstrated positive outcomes in creating significant changes in healthy eating and/or physical activity [5]. In adolescents, school-based interventions have resulted in decreased sedentary behaviors and increased physical activity [6,7]. After-school programs, using SCT-based invention also showed improvements in nutrition behaviors such as intakes of fruits and vegetables, healthy snacks, water, and sugar-free beverages and physical activity among 8–13 year old children [8,9]. Outside of the school environment, significant behavioral improvements were also observed among preadolescents and adolescents after the completion of SCT-based nutrition and/or physical activity interventions [10-12].

Several existing survey instruments on nutrition and physical activity primarily measure knowledge and behavior [13-16], but few assess self-efficacy which is a significant component of the SCT [17]. The most cited self-efficacy items for nutrition interventions among elementary school children were created by the Gimme 5 program [14] and “Smart Bodies” [18]; however, both programs exclusively focused on fruits and vegetables. In addition, the self-efficacy component from the After-School Student Questionnaire, modified from the Health Behavior Questionnaire, focuses only on dietary behaviors related to sodium and fat intake [15,19].

With the increasing need for theory-based intervention programs that target behavior changes [20], a valid and useful measurement tool is needed to assess the impact of SCT-based nutrition interventions on the main constructs of the theory, particularly self-efficacy. Contento et al. conducted a review of instruments for nutrition education programs, suggesting that the measurement tools should reflect the study design, intervention, and objectives while still having substantial validity and reliability [21]. Furthermore, according to the SCT, knowledge of health risks and benefits is a construct that creates the precondition for change [17,22]. Behavioral capacity, another construct of the SCT, is composed of the knowledge and skills necessary to perform a health behavior [23,24]; however, beliefs of self-efficacy are needed for most people to overcome the barriers to adopting and maintaining healthy lifestyle habits [17]. Therefore, the purposes of this pilot study were: 1) to develop and validate a SCT-based survey instrument that focuses on knowledge, behavior, and self-efficacy for fifth grade students; 2) to assess the relationships between knowledge, behavior, and self-efficacy, the main constructs of the SCT; and 3) to assess knowledge, behavior, and self-efficacy for healthy eating among the fifth grade students.

## Methods

### Participants and procedures

This investigation was approved by the University of Nebraska Internal Review Board. A total of 98 fifth grade students (aged 9–12 years) were recruited from four local elementary schools which had never been exposed to any supplementary nutrition education curriculum and had various numbers of students receiving free and reduced price meals (6.5-47.3%). Surveys were administered in the students’ regular classrooms. Students were directed to answer each question to the best of their ability, and if at any point they did not understand a word or question or what a question was asking, or were confused for any other reason, to circle what was confusing on the survey and write why it confused them. Parent consent and youth assent for each participant were obtained before the data collection.

### Survey instrument construction and scoring

The current instrument, The Healthy Habits Survey, was developed to assess the long-term impact for Growing Healthy Kids (GHK), a SCT-based, nutrition and physical activity curriculum for elementary students. The instrument utilizes the constructs of knowledge, behavior and self-efficacy with topic areas including digestion, physical activity, healthy meals, healthy snacking, food groups, breakfast, and meal planning, which were applicable to material covered in GHK. Items on the survey were selected or modified from the following programs or instruments: KidQuest [25], Network for a Healthy California Youth Survey [26], Nutrition Education Program [27], SIRK [26], CATCH [13], and PizzaPlease [28]. Certain knowledge questions and all self-efficacy questions were created as existing knowledge and self-efficacy items did not address topics relevant to GHK. The combination of existing and new items generated a 77-item instrument. The reading level was found to be appropriate for the fifth grade level based on a combination of common readability indicators [29].

For behavior questions, the responses to the items were scored from 1 to 4 (or 1 to 5 if there were 5 responses to the question) with a higher score reflecting a more positive response. Items were reversely scored when questions were related to an unhealthy behavior. Similarly, items for self-efficacy were scored from 1 to 3, indicating low, medium, and high self-efficacy, respectively. For each of the knowledge items, “1” was given if the participant had the correct answer, and if not, “0” was marked for the item.

### Content validation of survey instrument

Content and face validation [30] were used to validate the survey instrument. Initially, the survey included 77 items focusing on knowledge, behavior, and self-efficacy. Two content experts reviewed and reduced the survey to 68 items. The survey was then validated using an additional nine nutrition experts (content validation) and three lay experts (face validation). The nine content experts were Extension educators, assistants, and program leaders involved with implementing school-based nutrition and physical activity curriculum, of which five were Registered Dietitians and seven had Master degrees or higher.

For content validation, all experts received a cover letter, an instruction sheet, and a draft of the survey instrument. The cover letter provided an overview and the purpose of the study, asking each expert for their input on the survey. Experts were asked to independently rate each survey item on a 1 to 4 scale based on two validity factors: relevance and clarity [30]. Relevance referred to the item’s ability to represent the lessons covered through GHK (1 = the survey item is not representative, 2 = major revisions are needed to be representative, 3 = minor revisions are needed to be representative, 4 = the survey item is representative). Clarity represented how clearly the item was worded (1 = the item is not clear, 2 = major revisions are needed to be clear, 3 = minor revisions are needed to be clear, 4 = item is clear). Experts were also asked to provide additional comments addressing repetition, difficulty, appropriateness to income level, what was unclear about any question, and general suggestions they had on each item. An average rating of relevance and clarity for each item was calculated. Items scoring less than 3.0 in the relevance or clarity category were removed from the instrument. Items scoring between 3.0 -4.0 were either removed or edited based on handwritten comments from the experts. Face validity was conducted among three fifth grade teachers (lay experts) who gave qualitative feedback on the survey.

### Data analysis

For quantitative validation data, mean relevance and clarity scores were calculated respectively, based on the nine experts’ average ratings. The internal consistency/reliability for each section (knowledge, behavior and self-efficacy) was analyzed using Cronbach’s alpha with a value of 0.60 or higher deemed as acceptable [21]. Qualitative data for content validation and pilot testing were transcribed, coded, and grouped into reoccurring comments or themes for each item identified.

The scores of each participant’s responses to self-efficacy questions regarding healthy eating in the survey were summarized and the median value was identified by ranking all of the participants based on their summary scores (total self-efficacy scores). After excluding individuals with the median value of summary self-efficacy scores (n = 19), the remaining participants were stratified into “high self-efficacy” and “low self-efficacy” groups based on the following criteria: high self-efficacy group, summary self-efficacy scores > median value; low self-efficacy group, summary self-efficacy scores < median value. In addition, each subject’s scores for healthy eating related behavior questions and knowledge items were totaled. Differences in means of summary and individual behavior scores and summary knowledge scores between the two groups (high self-efficacy vs. low self-efficacy group) were examined using t-test. The relations between behavior, self-efficacy, and knowledge summary scores were assessed using Pearson Correlation Coefficient.

Due to the multi-dimensional nature of the constructs, factor analysis was used to identify underlying main factors/patterns associated with behavior or self-efficacy variables, with the assumption that participants responded similarly to certain questions which are all associated with a latent variable (factor). SPSS 22 (SPSS, Inc, Chicago, IL) was used for all statistical analyses with a two-sided *p* value of <0.05 considered statistically significant.

## Results

### Content and face validity

Qualitative comments from the nine experts are presented in Table 1. Four items in the relevance category had an average score of <3.0 and were removed from the scale. Among the 64 items that scored between 3.0 - 4.0 in the relevance category, 38 were removed and 17 were edited based on handwritten comments addressing repetition, difficulty, appropriateness to income level, and general suggestions. Seven items in the clarity category had an average score of <3.0 and were removed from the scale (three of which had already been targeted for removal due to low relevance scores). Sixty-one items had scores between 3.0 - 4.0 and were edited based on comments indicating what was unclear about each question. Seven additional items were added to compensate for removed items. The final survey instrument used in pilot testing contained 37 items including sections of demographics (4), knowledge (11), behavior (12), and self-efficacy (10). Results reported in the current study for our pilot participants were based upon this 37-item survey. For face validity, the qualitative feedback from the three fifth grade teachers varied; however, the disagreements appeared to focus on the knowledge questions and were not consistent enough to warrant further editing (Table 1).

**Table 1:** **Summary of comments from content experts (n = 9) for content validity and lay experts (n = 3) for face validity**

**Themes**	**Quotes**
**Content validity results (n = 9)**	
Repetition	“Question may not be necessary.”
	“Only need 1–2 of these types of questions.”
	“Repetitive.”
Difficulty	“Whole grains are sometimes hard for them to understand.”
	“They won’t know what these are.”
	“These choices will be a bit confusing.”
Appropriateness to income level	“Our students do skip meals but not because they want to, because they do not have food.”
	“This is the parent’s responsibility not the child’s.”
	“I think #12 may make some students feel bad or sad that they do not participate especially if it is because of money. It may not be a choice for the kids.”
	“This question could make students feel bad about being in a low income family.”
**Face validity results (n = 3)**	
Too Difficult	“I couldn’t answer some of these vitamin questions myself.”
	“I don’t know if students can answer all of these questions [pointing to vitamin questions].”
	“We don’t teach all of these topics here, so they probably won’t know some of this stuff.”
Appropriate for grade level	“These all look good.”
	“All of these are appropriate for the grade level.”
	“I don’t think students would have difficulty with any of these questions.”

Note: Content validity was conducted using 9 content experts and face validity was conducted using 3 5^th^ grade teachers (lay experts).

### Pilot testing and internal consistency

All fifth grade students (n = 98) completed the survey instrument, which took approximately 30 minutes. Cronbach’s alpha for knowledge was 0.41 and increased to 0.56 after removing items with low reliability. Qualitative results from student feedback showed difficulty in understanding words such as “carbohydrate” and “vitamin” although these items were validated at a fifth grade level. Therefore, knowledge items were primary replaced with alternate published items at a lower level of difficulty [25,27,28], but still rated at a fifth grade level.

Cronbach’s alphas for behavior and self-efficacy were 0.60 and 0.67, respectively. The corresponding values were increased to 0.71 and 0.70 when the items with low reliability were excluded. Removal of some behavior questions necessitated the addition of published beverage items [26] to address topics that were eliminated due to low reliability. This led to a final, fully developed survey instrument (which will be used to assess the Growing Healthy Kids nutrition education curriculum) that included 40 items with basic demographics (3 items) and knowledge (14 items), behavior (12 items), and self-efficacy (11 items) assessing the following topics: healthy meals, food groups, healthy snacking, healthy beverages, physical activity, breakfast, daily recommendation, and meal planning. The added items (e.g., knowledge items on food groups and daily recommendations) in the final survey were not re-tested since they were validated previously. Furthermore, notes from qualitative observations while students completed the survey indicated that 1) Students rushed to complete survey; 2) students failed to complete the entire survey; or 3) Students had difficulty with demographic questions.

### Demographics, behaviors, self-efficacy, and knowledge

Among 98 fifth grade students from four schools who completed the survey, 40% were males and 60% were females. The majority of students (56%) identified that they were not Hispanic/Latino and 37% identified that they were white. However, relatively high numbers of students did not know whether they were Hispanic (36%) or their race/ethnicity (23%) (Table 2).

**Table 2:** **Demographics and healthy eating behavior, self-efficacy, and knowledge of study participants (the fifth grade students, n = 98)**

**Demographics**	**N (%)**
Gender	
Male	39 (40)
Female	59 (60)
Race/ethnicity	
American Indian or Alaska Native	5 (5)
Asian	9 (9)
Black or African American	3 (3)
White/Caucasian	36 (37)
Two or more races	9 (9)
Other, not listed	14 (14)
I don’t know	22 (23)
Hispanic/Latino	
Yes	8 (8)
No	55 (56)
I don’t know	35 (36)
Behavior*	
Eat fruits	
None	26 (27.4)
1 time/day	34 (35.8)
2 times/day	14 (14.7)
3 or more times/day	21 (22.1)
Eat vegetables	
None	27 (28.4)
1 time/day	37 (39.0)
2 times/day	21 (22.1)
3 or more times/day	10 (10.5)
Eat whole grains	
None	4 (4.3)
1 time/day	21 (22.3)
2 times/day	30 (31.9)
3 or more times/day	39 (41.5)
Eat lean protein	
None	9 (9.6)
1 time/day	28 (29.8)
2 times/day	34 (36.2)
3 or more times/day	23 (24.5)
Eat/drink dairy foods/drinks	
None	3 (3.2)
1 time/day	18 (19.0)
2 times/day	39 (41.1)
3 or more times/day	35 (36.8)
Eat French fries or chips	
None	57 (60.0)
1 time/day	22 (23.2)
2 times/day	12 (12.6)
3 or more times/day	4 (4.2)
Drink sweetened beverages (pop, punches, sport drink, etc.)	
None	56 (59.)
1-2 time/day	33 (34.7)
3-4 times/day	5 (5.3)
5 or more times/day	1 (1.1)
Eat doughnuts, cookies, brownies, cakes, candy	
None	32 (33.7)
1-2 time/day	52 (54.7)
3-4 times/day	8 (8.4)
5 or more times/day	3 (3.2)
Eat breakfast	
0 days/week	2 (2.1)
1-2 days/week	5 (5.3)
3-4 days/week	6 (6.4)
5-6 days/week	23 (24.5)
7 days/week	58 (61.7)
Help plan family meals at home	
0 days/week	19 (20.2)
1-2 days/week	27 (28.7)
3-4 days/ week	30 (31.9)
5-6 days/week	6 (6.4)
7 days/week	12 (12.8)
Self-efficacy* ^**†**^	
Identify a healthy meal	
High	55 (56.7)
Medium	40 (41.2)
Low	2 (2.1)
Choose a healthy meal at home	
High	61 (62.9)
Medium	33 (34.0)
Low	3 (3.1)
Choose a healthy meal at school	
High	53 (55.2)
Medium	39 (40.6)
Low	4 (4.2)
Choose a healthy meal when your friends do not	
High	34 (35.8)
Medium	54 (56.8)
Low	7 (7.4)
Choose a meal with all five food groups	
High	32 (33.0)
Medium	55 (56.7)
Low	10 (10.3)
Plan a meal with at least three different food groups	
High	63 (65.0)
Medium	29 (29.9)
Low	5 (5.1)
Choose a healthy snack	
High	64 (66.7)
Medium	30 (31.3)
Low	2 (2.1)
Eat breakfast every morning	
High	62 (64.6)
Medium	28 (29.2)
Low	6 (6.3)
Knowledge*	% of the participants answered correctly
Healthy snack	92.9
Healthy meal	79.6
Healthy breakfast	99.0
Healthy beverage	99.0
Digestion	81.4
Nutrients like carbohydrate	55.1
Nutrients like protein	85.6
Nutrients like calcium	82.3
Nutrients like vitamin A	14.9
Nutrients like vitamin C	18.5

*1 to 9 participants had missing data on the behavior, self-efficacy, or knowledge variables.

^†^High self-efficacy, very sure; medium self-efficacy, somewhat sure; low self-efficacy, not sure at all.

Two thirds of the participants reported that they ate fruits (63.2%) or vegetables (67.4%) less than twice per day with approximately one third indicating no consumptions of either fruits (27.4%) or vegetables (28.4%). However, the majority stated that they ate whole grain (73.4%) or lean protein foods (60.7%), or ate/drank dairy products (77.9%) at least two times per day, with over one third consuming whole grains (41.5%) or dairy (36.8%) at least three times per day. Almost two thirds reported that they ate breakfast every day (61.7%) and 59.0% said that they did not drink any sweetened beverages such as pop, punches, sport drink, or fruit flavored drink. Over 50% (51.1%) of the participants reported that they helped plan family meals at home at least 3 days per week. Self-efficacy scores trended to be high, with 89.7% to 98.0% of the participants having either high (very sure) or medium levels (somewhat sure) of self-efficacy on the relevant variables. Greater than 90% of the participants answered correctly on knowledge questions regarding healthy snack, healthy meal, healthy breakfast and healthy beverage; however, a majority of the participants had difficulties in questions on specific nutrients such as vitamin A (14.9% scored correctly) and vitamin C (18.5% scored correctly) (Table 2). Since our interest was to assess healthy eating behavior, self-efficacy, and knowledge among our study participants, results of physical activity related items (2 in behavior, 2 in self-efficacy, and 1 in knowledge section) were not included in the current study.

Table 3 demonstrates behavior and summary knowledge scores based on the self-efficacy profiles. Compared to the low self-efficacy group, the high self-efficacy group had significantly higher scores on eating fruits (P = 0.0009), consuming dairy products (P = 0.009), eating breakfast (P = 0.008) and helping plan family meals at home (P = 0.0006), and higher summary behavior scores on healthy eating (P = 0.001). There were no differences in summary scores of nutrition knowledge between the two groups (P = 0.74). Summary scores of self-efficacy and behavior were positively correlated (r = 0.40, P = 0.0001); however, summary knowledge scores were not associated with self-efficacy (r = 0.02, P = 0.88) or behavior summary scores (r = 0.14, P = 0.23).

**Table 3:** **Scores of heathy eating behaviors and nutrition knowledge based on self-efficacy profiles of study participants (the fifth grade students)**

	Scores* (mean ± SD)		
	Low self-efficacy*	High self-efficacy*	**P value** ^**†**^
	**(n = 38)**	**(n = 39)**	
Summary self-efficacy^‡^	11.00 ± 0.91	15.89 ± 2.00	<0.0001
Behavior			
Eat vegetable	2.08 ± 1.02	2.23 ± 0.96	0.50
Eat fruits	1.94 ± 1.01	2.76 ± 1.06	0.0009
Eat whole grains	3.13 ± 0.84	3.21 ± 0.86	0.71
Eat lean protein	2.62 ± 1.01	2.92 ± 0.77	0.15
Eat dairy	2.97 ± 0.88	3.44 ± 0.60	0.009
Drink less sweetened beverages (soda, punches, etc.)	3.61 ± 0.59	3.41 ± 0.72	0.20
Eat less French fries or chips	3.50 ± 0.73	3.33 ± 0.90	0.37
Eat less donuts, cookies, brownies, cakes, candies	3.08 ± 0.75	3.33 ± 0.62	0.11
Eat breakfast	4.08 ± 1.22	4.68 ± 0.57	0.008
Help plan family meals at home	2.16 ± 1.08	3.10 ± 1.21	0.0006
Summary behavior^‡^	32.75 ± 4.17	35.92 ± 3.75	0.001
Summary nutrition Knowledge^‡^	7.84 ± 1.37	7.74 ± 1.33	0.74

*The scores of each participant’s responses to healthy eating related self-efficacy questions were summarized and the median value of the summary scores for the participants was obtained; Low self-efficacy participants were those with self-efficacy summary scores < the median value and high self-efficacy participants were those with self-efficacy summary scores > the median value. Participants with the median value of self-efficacy summary scores were not included (n = 19).

^†^P values for differences between participants in high and low self-efficacy groups by t test.

^‡^Summary self-efficacy = summary scores of self-efficacy items related to healthy eating; Summary behavior = summary scores of behavior items related to healthy eating; Summary nutrition knowledge = summary scores of nutrition knowledge items.

Factor analysis results for healthy eating related behavior and self-efficacy variables are shown in Table 4. It appeared that the behavior or self-efficacy construct each was associated with three main underlying factors: consuming healthy foods (Factor 1), consuming unhealthy foods (Factor 2), and eating breakfast and eating lean protein (Factor 3) for behavior; identifying/choosing healthy meals and snacks (Factor 1), planning/choosing a meal with different food groups (Factor 2), and eating breakfast every morning and choosing healthy meals at school (Factor 3) for self-efficacy. For behavior, Factor 1 of consuming healthy foods captured most of the variance (22.07%), and eating fruits (factor loading, 0.74) and vegetables (factor loading, 0.76) had the strongest associations with this latent variable (Factor 1). Factor 1 was also highly associated with consuming dairy products (factor loading, 0.63) and eating whole grains (factor loading, 0.53). In addition, drinking sweetened beverages (factor loading, 0.74) and eating French fries or chips (factor loading, 0.72) were strongly correlated to Factor 2 of consuming unhealthy foods. With respect to self-efficacy, a higher portion of the variance was explained by Factor 1 of identifying/choosing healthy meals and snacks (27.53%). Choosing a healthy meal at home (factor loading, 0.79) and choosing a healthy meal when your friends do not (factor loading, 0.79) had the strongest correlations with this factor.

**Table 4:** **Factor analysis for healthy eating behavior and self-efficacy among study participants (the fifth grade students, n = 98)**

	**Factor 1** ^**†**^	**Factor 2** ^**†**^	**Factor 3** ^**†**^
**Behavior variables**			
Factor loading*			
Eat vegetable	0.76	−0.07	0.09
Eat fruits	0.74	−0.01	0.30
Eat whole grains	0.53	0.11	0.35
Eat lean protein	0.35	0.05	0.65
Eat/drink dairy	0.63	0.32	−0.08
Drink sweetened beverages (soda, punches, etc.)	0.22	0.74	−0.28
Eat French fries or chips	0.03	0.72	0.16
Eat donuts, cookies, brownies, cakes, candies	−0.07	0.65	0.47
Eat breakfast	0.09	0.03	0.67
Variance explained			
Total	1.99	1.60	1.41
% of variance	22.07	17.78	15.76
**Self-efficacy variables**			
Factor loading*			
Identify a healthy meal	0.68	−0.04	0.21
Choose a healthy meal at home	0.79	0.15	0.01
Choose a healthy meal at school	0.38	0.20	0.62
Choose a healthy meal when your friends do not	0.79	0.05	−0.06
Choose a meal with all five food groups	0.20	0.69	0.25
Plan a meal with at least three different food groups	−0.01	0.82	−0.23
Choose a healthy snack	0.57	0.36	0.08
Eat breakfast every morning	−0.10	−0.12	0.84
Variance explained			
Total	2.20	1.35	1.25
% of variance	27.53	16.86	15.60

*Factor loading: Correlation of each behavior or self-efficacy variable with each factor using factor analysis. Higher absolute values represent higher correlations; “1” or “-1” represents the maximal correlation strength.

^†^For behavior: Factor 1 = consuming healthy foods, Factor 2 = consuming unhealthy foods; Factor 3 = eating breakfast and consuming lean protein; for self-efficacy, Factor 1 = identifying/choosing healthy meals and snacks, Factor 2 = choosing/planning a meal with different food groups, Factor 3 = eating breakfast every morning and choosing healthy meals at school.

## Discussion

Results from this study indicate that the Healthy Habits Survey is both a valid and useful tool to measure knowledge, behavior, and self-efficacy for SCT-based nutrition education programs among 9–12 year old students. Our study also suggested that participants with higher self-efficacy scores were more likely to report healthful eating behaviors.

The unique aspect of this survey instrument is the self-efficacy component. Self-efficacy is one of the important constructs of the SCT and limited surveys on nutrition education programs address this issue. Even within the existing instruments, none have assessed self-efficacy for a wide array of nutrition-related topics among children ages 9 to 12 years. Gimme 5, a SCT-based curriculum focusing on fruits and vegetables evaluated the impact on self-efficacy. The 22-item survey instrument, created for the target population of third to fifth grade students had an average alpha reliability of 0.90, but it only focused on assessing self-efficacy for eating fruits and vegetables [14]. “Smart Bodies” is another educational intervention based upon the SCT, targeting fourth and fifth grade students [18]. Similar to Gimme 5, the survey instrument demonstrated high alpha reliabilities for pre (0.92) and post lessons (0.90), but also had its main focus on fruits and vegetables [18]. The Coordinated Approach to Child Health (CATCH) study utilized modified items for self-efficacy from the Health Behavior Questionnaire; however, these items focus more on salt and fat intake and physical activity rather than a broad array of items [13]. In addition, these studies did not assess the long-term impact of the respective programs [13,14,18]. The Integrated Nutrition and Physical Activity program conducted a long-term follow-up of second grade students in the fifth to eighth grade, examining self-efficacy on food preparation and identification of foods with fat and found alpha reliabilities ranging from 0.72-0.75 [31], which were similar to our results (Cronbach’s alpha: initial survey = 0.67; revised survey = 0.70). The reliability results (Cronbach’s alpha: initial survey = 0.60; revised survey = 0.71) for the behavior section were consistent with what we hypothesized since all of the items in this section were taken from existing, validated instruments [25,26].

The internal consistency/reliability of knowledge questions appeared to be lower than hypothesized (Cronbach’s alpha: initial survey = 0.41; revised survey = 0.56) since a majority of the knowledge questions were taken verbatim or modified from existing, validated instruments. There are several possible explanations. The difficulty level of the knowledge questions might be higher for the students who had not received supplementary nutrition education, which was the case for our pilot participants. Our study population and the populations in the original studies from which these questions were taken may have been inherently different in certain demographics such as race/ethnicity, gender, and socioeconomic status. In addition, we measured a broad range of knowledge including knowledge necessary to conduct nutrition related health behaviors and knowledge of certain nutrients, such as carbohydrates and vitamins. The reasons for including the measures of the more specific nutrition knowledge were: 1) knowing the benefits of vitamins and carbohydrates, for instance, would demonstrate the importance for children to eat a healthy and balanced meal; and 2) our knowledge scale was created for assessing GHK where health benefits of carbohydrate and vitamins were taught in the curriculum.

In our study, self-efficacy and behavior (for healthy eating) summary scores were positively associated; however, there were no associations of knowledge scores with behavior or self-efficacy. The current results demonstrated that participants with high self-efficacy also had higher behavior scores (eat fruits, eat/drink dairy products, eat breakfast, help plan family meals at home, and summary behavior scores) than those with low self-efficacy, suggesting that self-efficacy may be more relevant than knowledge in terms of influencing children’s eating behaviors. However, the low reliability of Cronbach’s alpha for knowledge may have influenced the relationship between knowledge and behavior. Our findings were consistent with the SCT, which suggests that self-efficacy plays an important role in an individual’s behavioral changes [17]. Indeed, Ramirez et al. found self-efficacy to be a predictor of physical activity in fourth to sixth grade students [32]. Results from Farm to School programs have also demonstrated an association between behavior and self-efficacy in fourth to sixth grade students based on self-report data [33]. Factor analysis results suggest that consuming healthy foods and choosing/identifying healthy meals and snacks were key underlying factors for behavior and self-efficacy constructs, respectively. It also suggested that eating fruit and vegetables was more relevant in terms of the consumption of healthy foods.

The observed significant differences in behavior variables regarding fruit or dairy intakes, and eating breakfast between participants with high and low self-efficacy scores may be explained in part by the ongoing efforts made by the schools in concert with several national programs such as the United States Department of Agriculture’s Fresh Fruit and Vegetable Program [34], the Healthy, Hunger-Free Kids Act of 2010 [35], and the National School Lunch and School Breakfast Programs [36]. These efforts increase the accessibility of fruits, dairy foods, and breakfast in the school environment, thereby raising self-efficacy of improving these eating behaviors among the students, leading to enhanced performance of target behavior. However, it appeared that self-efficacy did not influence students’ vegetable intake even though making fresh vegetables more accessible was also part of the efforts from some of these programs [34,35]. It is possible that the taste of fresh fruits was more appealing to children as compared to that of fresh vegetables. Future studies with a larger sample size are necessary to confirm our findings.

Two thirds of the fifth grade students in our study reported that they either ate fruits or vegetables only once or did not eat them at all on a typical day, with approximately half indicating no consumptions of any of these foods. Although the current results need to be further confirmed in the studies in which the participants are randomly drawn from a large population, they in general, reflect a pattern of dietary behaviors among children in the Midwestern area since the four elementary schools involved in our study had similar demographics (i.e., race/ethnicities, socioeconomic status of the schools) compared to the overall student population in the area. Our findings that a relatively high proportion of the fifth grade students tended to consume less or no fruits or vegetables suggest that strategic interventions are needed to address this ongoing problem among elementary school children.

The current investigation is the first study which developed and validated a survey instrument that includes self-efficacy assessment for an educational program covering a variety of nutrition-related topics for older elementary school students (9 to 12 years). There are several strengths of the study. The study population was diverse in terms of race/ethnicity and school socioeconomic status, providing a wide range of perspectives that reflect both the Title I (≥40% students receiving free or reduced price school meals) and non-Title I (<40% students receiving free or reduced price school meals) schools that this instrument will be used to evaluate in the future. The survey instrument developed in our study was validated using various methods, including content validity, face validity, and internal consistency reliability. Furthermore, the constructs of the survey were strengthened by the significant correlation between behavior and self-efficacy scores and the factor analysis outcomes which identified the key patterns related to healthy eating behaviors and self-efficacy. Additionally, the inclusion of measurements of behavior and self-efficacy and the necessary knowledge to perform nutrition related health behaviors would allow this instrument to apply to many SCT-based nutrition programs beyond GHK; however, as with many surveys, it may need to be modified to fit into each individual program and the specific population associated with the program.

Our study has limitations. The participants (fifth grade students) were recruited from schools that had never been exposed to any nutrition interventions. Therefore, the pilot testing results, particularly for knowledge, may not be generalized among other fifth grade students who have received supplementary nutrition education. Also, due to the nature of convenience sampling, our findings regarding children’s dietary behaviors may not completely represent the entire fifth grade student population; nevertheless, the current study population varied in race/ethnicity and socio-economic status with a range of 6.5% to 47.3% of the students among the four schools involved receiving free or reduced price school meals. Furthermore, although our measures were validated, additional assessments such as food diaries/records or behavioral observation may help provide a more accurate evaluation of behaviors and reduce self-report/response bias, particularly among children. There may also have been issues with common method variance, though we employed different means to minimize this type of bias, including the use of different scale types for different constructs, incorporation of both negative and positive behavior items, use of familiar survey format, and assurance of anonymity to encourage truthful answers. Lastly, according to the qualitative results, children had difficulty with demographic questions and the feeling of being rushed and missing items, leading to potential information bias. For future implementation of this instrument among children of this age group, we recommend to simplify demographic items and remind students to check every page of their surveys when finishing so that all the survey questions will be completed.

## Conclusion

The current results indicate that the Healthy Habits Survey is a valid and useful tool to evaluate the effectiveness of SCT-based nutrition education programs that teach broad knowledge of nutrition and physical activity among older elementary school students. In addition, our results suggest that participants with higher self-efficacy scores were also associated with higher behavior scores for healthy eating. However, the fact that relatively high proportions of the fifth grade students in our study had low intakes of fruits or vegetables warrants strategic interventions to facilitate the behavior change.
